# MINDSET 2.0: feasibility of digital decision support for epilepsy self-management

**DOI:** 10.3389/fneur.2026.1913366

**Published:** 2026-09-23

**Authors:** Refugio Sepulveda, Ross Shegog, Katarzyna Czerniak, Robert Addy, Kimberly Martin, Tomas Nuño, Sheila Soto, Abdul Tawab Saljuqi, Ramses Sepulveda, David Labiner

**Affiliations:** 1 Mel and Enid Zuckerman College of Public Health, University of Arizona, Tucson, AZ, United States; 2 Department of Health Promotion and Behavioral Science, UTHealth School of Public Health, Houston, TX, United States; 3 Epilepsy Foundation Central & South Texas, San Antonio, TX, United States; 4 Department of Neurology, College of Medicine, University of Arizona, Tucson, AZ, United States

**Keywords:** clinical decision support, digital health, epilepsy comorbidities, epilepsy self-management, feasibility study, quality of life, social determinants of health

## Abstract

**Background:**

The Managing Epilepsy Well Network (MEWN) epilepsy self-management (ESM) training programs assist people with epilepsy (PWE) in improving their ESM and co-morbidities. The online bilingual Management Information & Decision Support Epilepsy Tool (MINDSET) was enhanced to provide recommendations for these programs and to assess non-medical drivers of health (NMDH).

**Purpose:**

This study aimed to assess the feasibility of MINDSET 2.0, guided by Bowen et al.’s feasibility framework, and its perceived efficacy in providing PWE and healthcare providers with a tailored goal-based action plan, including ESM program recommendations and identified NMDH.

**Methods:**

A single-group feasibility trial was conducted from March to August 2022 with seven adult PWE in Arizona (*n* = 4) and Texas (*n* = 3) who completed MINDSET 2.0 before their regular clinic visit, discussed their action plan with their neurologist, and then completed a feasibility rating scale and exit interview.

**Results:**

Participants rated MINDSET 2.0 as acceptable, likable, credible, easy to use, appealing (100% agreement), of appropriate duration (83%), easy to understand (67%), impactful for managing epilepsy (75–100%), and found the program helpful (100%). The MINDSET 2.0 recommendation algorithms demonstrated 100% agreement between programmed and generated recommendations, confirming that the software executed the predefined decision rules as intended. Clinical feasibility of MINDSET 2.0 was evaluated separately through usability, acceptability, and implementation-related measures. Three participants needed help using MINDSET 2.0 (50%). Neurologists (*N* = 3) rated MINDSET 2.0 as increasing the thoroughness of information collected from and improving communication with PWE.

**Conclusion:**

In addition to generating an ESM action plan, MINDSET 2.0 may be feasible in assisting PWE and neurologists in decision-making regarding the most suitable MEWN ESM training programs and the most pressing NMDH. Given that no participants reported prior experience with MEW programs, these ratings likely reflect participants’ familiarity with and initial impressions of the recommended interventions rather than direct evaluations of their value or effectiveness. Further efficacy testing is required.

## 1 Introduction

Epilepsy self-management (ESM) comprises a range of behaviors that people with epilepsy (PWE) practice to control their disorder, including (1) medication management (e.g., adherence to prescribed medication and clinical visit regimens), (2) seizure management (e.g., preparation for and response to seizure episodes), and (3) lifestyle management (e.g., altering behaviors to avoid seizures and/or adverse consequences) (1).

There is growing evidence of an association between ESM and seizure control, quality of life, and comorbidities of depression and memory deficits (2, 3). The Managing Epilepsy Well (MEW) Network is devoted to the research and development of programs to improve self-management among PWE. MEW 2.0 represents a recent initiative to broaden empirical evidence on the effectiveness of these programs through replication. The evidence base for MEW programs to improve ESM and mitigate associated comorbidities is increasing (4). Evidence-based MEW programs include PACES, UPLIFT, and HOBSCOTCH. Project PACES (Program of Active Consumer Engagement in Self-management) increases the medical, psychosocial, and community adjustment of adults with epilepsy; Project UPLIFT (Using Practice and Learning to Increase Favorable Thoughts) reduces depression by improving mindfulness and cognitive-behavioral skills; and Project HOBSCOTCH (Home-Based Self-management and Cognitive Training Changes Lives) improves memory and attention in PWE (5–11).

Emerging empirical evidence suggests that ESM programs can positively impact patients’ ESM and comorbidities and prevent breakthrough seizures, which can lead to increased hospital use and mortality (2). However, their broad adoption and implementation in clinic-based treatment programs remain a challenge. Barriers to implementation include awareness of the programs and matching patient needs to specific programs (12).

Non-medical drivers of health represent an array of environmental circumstances that can influence the ESM behaviors of PWE (13), and recommendations for their assessment are components of population health initiatives (14). Despite this, neurology clinics do not systematically collect PWE management and social determinant data, and few have decision-making protocols to guide treatment planning that can be informed by such assessments.

Protocols to screen and assess patients for clinical, co-morbidity, behavioral, and social needs on recommending MEW training programs that are tailored to patient needs may assist health care providers in improving ESM and mitigating the comorbidities of depression and cognitive deficits in patients. The Management Information & Decision Support Epilepsy Tool (MINDSET) is a bilingual (English and Spanish) ESM decision support program designed to enhance patient awareness of ESM behaviors and patient engagement with their Health Care Providers (HCPs) in improving behavior through a shared decision-making process (15–17). Patients select a language preference (English or Spanish) and enter their clinical and ESM data by responding to embedded validated scales (described in detail below). Patients select three ESM goals to improve seizure, medication, and lifestyle management, each tailored to the patient’s behavioral risk profile, choose strategies to accomplish these goals, and rate their self-efficacy in being able to achieve them by their next clinic visit. MINDSET compiles this data to provide a tailored goal-based Action Plan that assists patients and HCPs in discussing self-selected management goals to improve ESM. The feasibility and efficacy of MINDSET in identifying ESM behaviors and increasing ESM adherence in PWE have been described in previous studies (15–17). In response to the need for telemedicine and distance options for ESM training, MINDSET was modernized into an online, responsive, cross-platform application that is accessible on any device with a working internet connection (MINDSET 2.0). Enhancements include added assessment tools to assess the social determinants of health (HealthLeads), quality of life (QoLIE-10), and memory (QoLIE-31 cognitive subscale).

MINDSET 2.0 is designed to enable a systematic assessment within the clinic visit of a patient’s ESM behaviors, comorbidities (depression and memory), and social determinants that are perceived as most pressing, and then to provide feedback to the patient with a tailored referral to appropriate MEW ESM programs (based on the PWE’s profile and past program experience) for deeper intervention on ESM (PACES), depression (UPLIFT), and memory challenges (HOBSCOTCH). Figure 1 shows the design algorithms for patient assessment (18, 19). The structure, function, and usability testing of MINDSET 2.0 have been reported elsewhere (18). The purpose of this study was to test the feasibility and perceived efficacy of MINDSET 2.0 in providing a goal-based ESM treatment plan, assessment, and feedback on important social determinants, and recommendations for linking MEW programs tailored to patient needs.

**Figure 1:**
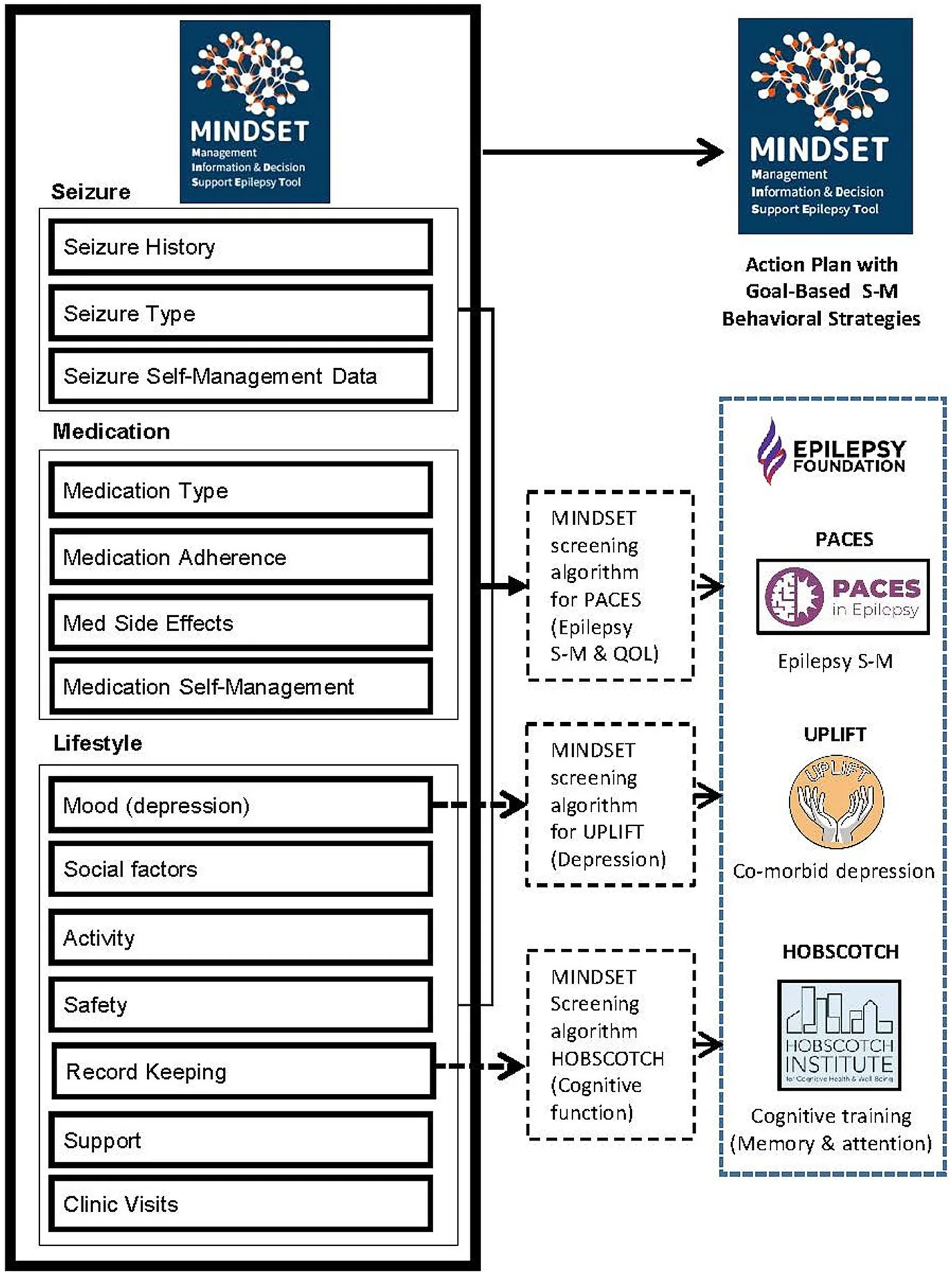
Design algorithms for patient assessment and linkage to MEW EBPs.

## 2 Methods

### 2.1 Study design

A single-group feasibility trial was conducted over 6 months from March to August 2022 with seven adult PWE in Arizona (*n* = 4) and Texas (*n* = 3) who used MINDSET 2.0 during their regular clinic visits. PWE were recruited from six neurology clinics in Texas and Arizona and completed MINDSET 2.0 during their clinic visits before a broader clinic-based randomized controlled trial (RCT). The study was approved by the human subjects’ institutional review boards of the University of Arizona College of Medicine and the University of Texas Health Sciences Center at Houston. This feasibility study was reported in accordance with the *Consolidated Standards of Reporting Trials* (CONSORT) extension for pilot and feasibility trials (20).

### 2.2 Clinic settings

The study was conducted at two neurology clinics in Arizona affiliated with The University of Arizona and the Banner University Medical Center—Tucson, and four clinics in Texas operated by the Epilepsy Foundation Central, South Texas (EFCST). The Banner Clinics serve a large number of Hispanic PWE, with approximately 30% preferring to speak Spanish during their clinic visits. The EFCST clinics located in Harlingen, Corpus Christi, San Antonio, and Uvalde mostly serve low-income and uninsured Spanish-speaking PWE (21). The Uvalde clinic was shut down for several months during the study due to the Uvalde Elementary School shooting in May 2022, preventing recruitment for feasibility testing.

### 2.3 Participants

The patient inclusion criteria comprised a diagnosis of epilepsy, age ≥ 18 years, English and/or Spanish-speaking, and willingness and ability to complete the study protocol. Patients were excluded if they had a diagnosis of any rapidly progressive neurological or medical disorder or other major neurological impairments, such as motor disorders (e.g., hemiplegia or quadriplegia), learning difficulties, and psychiatric/behavioral problems (e.g., autism and attention deficit disorder), which would inhibit their ability to use MINDSET 2.0, complete surveys, or practice ESM activities. Four neurologists, two each from Arizona and Texas, respectively, participated in the study, three of whom completed the MINDSET 2.0 rating scale.

### 2.4 Recruitment

Neurologists at each clinic identified patients scheduled for clinic visits who met the eligibility criteria and invited them to participate via letter. Interested patients were asked to call or return a signed ‘consent to contact’ form to permit contact by the research staff. If the form or phone call was received, the research staff contacted the patient by phone or conference call to explain the study and send consent documents for review. Patients had the option of in-person or remote participation, depending on their typical clinic visit arrangements. At the clinic visit study staff met the patient to obtain informed consent.

### 2.5 Study protocol

Before the first clinic visit, Neurologists were first given an orientation on how to use the MINDSET 2.0 Action Plan in the clinic encounter and guidance on how to reinforce and review this with the patient. Six patients were then recruited into the study in person at the clinics. After providing study consent, patients completed an online demographic questionnaire and received a brief orientation on how to use MINDSET 2.0. Research staff provided patients with a unique, non-identifiable 9-digit study PIN to use for logging into MINDSET 2.0. Research staff assisted participants as needed. Upon completion of MINDSET 2.0, patients were seen by their neurologist. During the clinic encounter, the patient and neurologist discussed the Action Plan. One participant completed the study remotely from home because their clinic was only operating remotely at that time (21). The participant used online links to complete consent forms, surveys, and MINDSET 2.0, and had a virtual telemedicine-mediated neurologist encounter. Following the clinic encounter, patients completed a program usability rating scale and an exit interview. At the end of the clinic visit, participants received a $30 incentive.

### 2.6 Feasibility framework and progression criteria

The feasibility evaluation of MINDSET 2.0 was guided by the framework proposed by Bowen et al., which identifies key feasibility domains including acceptability, demand, implementation, practicality, adaptation, integration, expansion, and limited efficacy testing (22). The current study focused primarily on acceptability, implementation, and practicality to evaluate whether MINDSET 2.0 could be integrated into routine neurology clinic workflows and whether progression to a future randomized controlled trial (RCT) was warranted.

Before study initiation, progression criteria were established to determine feasibility for a subsequent RCT. Criteria included: (1) ≥70% agreement between MINDSET 2.0-generated recommendations and the predefined recommendation algorithm to demonstrate successful software execution; (2) evidence of acceptability based on participant and provider feedback regarding usability and perceived value of the tool; and (3) evidence of practicality, including successful completion of the tool within the clinic workflow and without substantial disruption to routine care processes. Feasibility criteria were considered met if the predefined benchmarks were achieved, supporting advancement to a future RCT.

### 2.7 Measurement

#### 2.7.1 Participant demographics and epilepsy history

A 28-item survey assessed sex, age, ethnicity/race, length of residency in the United States, education level, marital status, income level, employment status, healthcare provider, access to technology (e.g., computers and smartphones), language preferences, quality of life, and epilepsy history (e.g., age at first seizure, age at diagnosis, general health status, and seizure frequency within the last 12 month and 30 day periods).

Five validated scales are embedded in MINDSET 2.0 to assess ESM adherence, depression, cognitive function (memory), QoL, and social determinants of health.

*ESM adherence*. An adapted version of the *38-item Epilepsy Self-Management Scale* assessed the frequency of ESM behavior across the domains of seizure, medication, information, safety, and lifestyle management using a 5-point Likert scale (Never to Always) (23, 24). Total scores are calculated by reverse coding the negatively worded items and summing the responses. Higher scores indicate more frequent use of ESM strategies. Low-frequency behaviors are flagged by MINDSET 2.0 as options for subsequent goal selection by participants.

*Depression*. The six-item *Neurological Disorders Depression Inventory for Epilepsy (NDDI-E)* Screening Tool assessed depression on a 4-point Likert scale (Never to Always or Often). The NDDI-E scores range from 6 to 24, with a score of 15 or higher indicating depression (25, 26). A score of 15 or higher initiates a recommendation in the Action Plan that the participant may be eligible for UPLIFT (10, 11).

*Cognitive function (memory).* The QOLIE-31 six-item cognitive subscale assesses cognitive function using a 4-point Likert scale. The possible final score range is 0–100, with higher scores reflecting better cognitive function. A score of 70 or lower initiates a recommendation in the Action Plan that the participant may be eligible for the HOBSCOTCH trial (7, 8, 18).

*Quality of Life.* The QOLIE-31 (Quality of Life in Epilepsy 31 survey) single overall QoL item uses a 10-point Likert scale (Worst Possible QoL to Best Possible QoL). Raw numeric values were converted to a 0–100 final point score, with higher scores reflecting better overall quality of life (27). The QOLIE-10 (Quality of Life in Epilepsy 10-item survey) assesses how often epilepsy affects health and daily activities in three domains: Epilepsy Effects (memory, physical, and mental effects of medication), Mental Health (energy, depression, general quality of life), and Role Functioning (seizure worry, work, driving, social limits) (28). Lower QOLIE-10 scores indicate fewer problems and a better QoL. A response indicating memory difficulties from QOLIE-31 initiates a recommendation in the Action Plan that the participant may be eligible for HOBSCOTCH (9).

*Social determinants*. The eight-item *Health Leads* Social Determinants Inventory assesses possible social barriers (non-medical drivers of health) to ESM, including factors such as income, education, housing, employment, food security, and neighborhood environment, allowing them to identify areas for targeted interventions to improve overall health outcomes (29). Each question was answered using a yes/no response option. A ‘Yes’ response for a determinant initiates the addition of that determinant to the Action Plan to cue neurologist-patient discussion for potential linkage to social services.

#### 2.7.2 Patient perceptions of MINDSET 2.0 acceptability

A 32-item usability survey was adapted to correspond with the MINDSET 2.0 functions for assessment of ease of use, likeability, credibility, ease of use, understandability, duration, usefulness, perceived impact, and motivational appeal and provided to participants after MINDSET 2.0 use (9, 17, 18). *Likeability* for the look and feel of the program and Action Plan (including color, buttons and sliders, font, and layout) was assessed on a 5-point Likert scale (dislike a lot’ to ‘like a lot’). *Credibility* was assessed on a 3-point Likert scale (can be trusted’, cannot be trusted’, and ‘do not know’). Ease was assessed for MINDSET 2.0 tasks including data entry, instruction and Action Plan clarity, goal selection, and strategy choice on a 4-point Likert response set (‘not easy, somewhat easy, very easy, do not know’), as well as a general question on whether MINDSET 2.0 was easy to use overall (‘yes, no, do not know’). *Understandability* was assessed using Yes/No response options for words used in MINDSET 2.0, and if help was required to complete it. *Duration* was assessed on whether the time taken to use MINDSET was ‘too long,’ just right’, or ‘too short’. *Usefulness* was assessed with a single item inquiring if MINDSET was more useful, as useful as, or less useful than other surveys or seizure programs used in the clinic. *Perceived impact* was assessed using four items with a 3-point Likert scale (yes, no, do not know) assessing the impact of MINDSET to encourage thought about ESM, aid in current and future epilepsy management, and aid in communicating with the health care provider. *Motivational appeal* was assessed on whether the participant would recommend MINDSET 2.0 to another patient or whether they themselves would use MINDSET 2.0 using a 3-point Likert scale (yes, no, do not know).

Exit interviews were conducted with patients at the end of the clinic visit, which comprised questions on what they liked the most and least about MINDSET 2.0, suggested enhancements, and what they would tell other patients about MINDSET 2.0.

#### 2.7.3 Neurologist perspectives on MINDSET 2.0 implementation

*Neurologist Demographics:* Demographic data from neurologists were collected using a 3-item healthcare provider profile survey on qualifications, total years in clinical practice, and years in their current position.

*Neurologist Feature Ratings:* An adapted 5-item feature checklist was used to assess neurologists’ perceptions of the impact of using MINDSET 2.0 against usual clinical practice (not using MINDSET 2.0) for managing patients with epilepsy. A 5-item semantic differential scale with a 5-point response set was used to assess perceptions of ease (easier/harder), thoroughness (more/less thorough), accuracy (more/less accurate), time (less/more time), and communication (improves/interferes with communication).

#### 2.7.4 Process measures

Incident logs were retained throughout the study to record any problems with using MINDSET 2.0, including program bugs, hardware failures, or patient difficulties with item responses. Issues were recorded and, if possible, rectified in real time. Research coordinators documented the time required for each participant to complete the MINDSET 2.0 program in the Incident Log during every study visit.

### 2.8 Analysis

Descriptive statistics were used to describe participant demographics, and mean item scores were reported for current ESM behavior adherence, depression, and quality of life parameters. Participant ratings of MINDSET 2.0 features were calculated as a percentage of participant agreement, and an *a priori* criterion of 70% agreement (i.e., that at least 70% of participants agree that a given MINDSET 2.0 component is acceptable, understandable, credible, etc.) was set as an *a priori* indicator of readiness for MINDSET 2.0 to be evaluated for efficacy. Descriptive statistics for neurologists’ ratings of MINDSET 2.0 features were calculated as well.

## 3 Results

### 3.1 Participant description

Seven patients participated in and completed the feasibility study. Four patients were recruited from Banner Neurology clinics in Arizona (57.1%) and three from EFCST clinics in Texas (42.9%). They self-reported as Hispanic (80%), 34.3 ± 10.9 years of age, and with an average residence in the U. S. of 29.6 ± 13.0 years (Table 1). They were mainly high school educated (42.9%), single (71.4%), employed for wages (57.1%), and most had health insurance coverage (57.1%), mainly Medicaid (28.6%) and private insurance (28.6%). Most were employed (57.1%) with income below $10,000 (57.1%) and had access to a smartphone (100%) and a computer (85.7%). Language preference when reading or speaking with friends and family was mostly English (28.6%), and when thinking, both Spanish and English equally (42.9%).

**Table 1:** Participant demographics (*N* = 7).

Construct	Category	n (%)
Location (State)	Texas	3 (42.9)
	Arizona	4 (57.1)
Age (yrs.)	Mean (±SD); Range	34.3 (±10.9); 18–49
Gender	Female	4 (57.1)
Ethnicity	Hispanic	6 (80.0)
Race^1^	White	5 (71.4)
Residency in U. S. (yrs.)	Mean (±SD); Range	29.6 (±13.0); 4–44
Education level	College 1–3 years	2 (28.6)
	GED (High School Graduate)	3 (42.9)
	Grade 11 or less (Middle / High School)	2 (28.6)
Marital status^2^	Single; Separated/divorced	6 (85.6)
	Married	1 (14.3)
Employment status^3^	Employed for wages	4 (57.1)
	Not working (Unable, unemployed, student)	3 (42.9)
Income level	Less than $10,000	4 (57.1)
	$10,000–$49,000	3 (42.9)
Insurance	Employer provided health insurance (Own plan)	2 (28.6)
	Medicaid	2 (28.6)
	No coverage	3 (42.7)
Technology access	Computer; Smartphone	6 (85.7); 7 (100.0)
Language preference (Reading & speaking with family and friends)	Both English and Spanish equally	2 (28.6)
	Only English	3 (42.3)
	Only Spanish	1 (14.3)
	More English than Spanish	1 (14.3)
Language preferences (Thinking)	Both English and Spanish equally	3 (42.9)
	Only English	2 (28.6)
	Only Spanish	1 (14.3)
	More English than Spanish	1 (14.3)

1 Missing response = 1(14.28%).

2 Categories of Living together/Common Law Married/ Widowed were not included.

3 Categories of Homemaker, Self-employed, Student, and Employed; Out of work for less than 1 year; Retired were not included.

Most participants (57.2%) reported that their health status was ‘very good’ (Table 2). The mean age of first seizure and of diagnosis with epilepsy were similar, ranging from less than 1 to 17 years. Four participants (57.14%) reported having at least one seizure in the past 12 months, and of those, three reported having at least one seizure within the last 30 days. Seizure frequency in the last 30 days ranged from 3 to 9 (median = 7), with an average of 6.33 (±3.06). Seizure frequency since last clinic visit ranged from 0 to 20 (average 4.67 ± 7.97). Participants missed an average of 0.16 (SD = 0.40) days of their AED during the previous 2 weeks, with a median of 0 days and a range of 0–1 day (Table 2). The average depression score on the NDDI-E was 10.57 ± 4.31 (range: 6–15), with one participant meeting the depression criteria (score≥15). The QoLIE-31 cognitive function subscale score averaged 68.33 ± 16.81, indicating memory problems during the past week.

**Table 2:** Participant medical parameters (*N* = 7).

Construct		Category	n (%)
Overall health	Health Status^1^	Excellent Very Good; Good Fair	1 (14.3) 4 (57.2) 2 (28.6)
Epilepsy Hx	Age at 1st seizure (years)^2^	Median; Range	1; <1–17
	Age at diagnosis (years)^2^	Median; Range	1; <1–17
Seizures	Seizure frequency^2^	Last 12 months	4 (57.1)
		Last 30 days	3* (24.6)
	Number of seizures in the last 30 days (days)^2^	Mean (±SD); Median; Range	6.33** (±3.06); 7; 3–9
	Seizure frequency since last clinic visit^1^	Mean (±SD); Median; Range	4.67 (±7.9); 0.5; 0–20
Medication	Number of days in the last 2 weeks that AED meds were missed^1^	Mean (±SD); Median; Range	0.16(±0.4); 0; 0–1
Depression	Mood (NDDI-E)^1^^,^^3^	Mean (±SD); Range Depression (Score ≥ 15)	10.57 (±4.3); 6–15 1
Cognitive Fn.	Cognitive Subscale (QoLIE-31)^1^^,^^4^	Mean (±SD)	68.33 (±16.81)
Quality of life	Overall Quality of Life (QoLIE-31)^1^^,^^5^	Mean score (±SD)	88.57 (±14.6)
	General Quality of Life (QoLIE-10)^1^^,^^6^	Mean score (±SD)	1.95 (±0.7)
Behavior	Epilepsy Self-Management^1^^,^^7^ Mean (SD)	Seizure	4.53 (±0.74)
		Medicine^8^	4.36 (±0.65)
		Lifestyle	4.12 (±0.61)
		Safety	4.27 (0.76)
		Information	3.42 (±0.54)
		Total Score^8^	4.14 (±0.41)
Social determinants	Health Leads^9^	Housing Instability	1 (14.3)
		Lack of Childcare	1 (14.3)
		Financial Resource Strain	2 (2.9)
		Transportation Challenges	1 (14.3)
		Social Isolation	1 (1.43)

1 Self-Reported in Mindset 2.0.

2 Self-Reported in Demographic Survey.

3 Responses, Likert scale — never: 1–always or often: 4, symptoms, maximum score = 24, score ≥ 15 is symptomatic.

4 Cognitive subscale (items 12, 15, 16, 17, 18, and 26) of the QOLIE-31. A combined score of ≥70 is obtained if the memory problem is “little” or “none” of the time.

5 Overall Quality of Life subscale (Item 1) of the QoLIE-31 scale. Higher scores reflect a better quality of life, whereas lower scores reflect a poorer quality of life.

6 QOLIE-10 scale. Patients with lowest scores have the least problems.

7 Self-management range of mean item scores from a 5-point Likert response set with 1-Never, 2-rarely, 3-Sometimes, 4-Most of the time, 5-Always (adapted from DiIorio ESM scale).

8 One item was removed from analysis due to missing data resulting from data collection difficulties.

9 Health Leads Social Determinants Inventory. Patients were able to select any that applied. The social determinants of food insecurity, utility needs, and health illiteracy were not included because none of the patients reported having issues in these areas.

*Four participants reported having had at least one seizure in the last 12 months. Of these, three reported having had at least one seizure in the last 30 days. **Of the three participants reporting at least one seizure in the last 30 days, the average number of seizures was 6.33 (±3.06).

QoLIE-31 scores averaged 88.57 ± 14.63, indicating a skew toward positive perceptions of QOL (Table 2). Participants’ ESM mean responses were positively skewed. Subscales ranged from 1.85 to 5.0. Mean item scores were higher for seizure (range 4.28–5.0) and medication management (range 4.0–4.57) subscales. Mean item scores for lifestyle, safety, and information management items ranged from 3.0 to 4.75, 3.42 to 4.6, and 1.85 to 4.4, respectively. Three participants self-reported having social determinants that impeded their ability to manage their epilepsy (range 1–3). The most selected was the inability to see a doctor because of financial issues (*n* = 2), then problems with housing, childcare, transportation, and social isolation (all *n* = 1).

### 3.2 Participant goal selection

ESM goals were unique to each participant, except for ‘always carrying an I.D. stating your epilepsy,’ selected by two participants (Table 3). Lifestyle management showed the highest frequency of risk behaviors (*n* = 5) compared to seizure and medicine management. Six of the seven participants rated their self-efficacy as “very sure” of meeting their goals.

**Table 3:** Most selected ESM goals/strategies and associated self-efficacy ratings (*N* = 7)^1^^,^^2^.

Risk behavior	Patient-selected goal	Most common strategies selected^3^
Seizure management (*n* = 2)^4^		
I never avoid seizure triggers.	Avoid seizure triggers, Identify seizure triggers.	Avoid noise or loud sounds
I rarely call my doctor if I am having more seizures than usual	Always contact your doctor or nurse when you are experiencing increased seizures.	Call your doctor’s office Keep a log of seizures
Medicine management (*n* = 3)^4^		
I do not use a pill container/pill box for my seizure medicines.	Use a pill container/pill box for my seizure medicines	Use a pill box
I rarely plan ahead so that I do not run out of my seizure medicine.	Plan ahead so that you always have enough medicine in stock.	Other “I’m trying to figure this crap out, but my pharmacy is very annoying.”
I never use reminders to take seizure medication.	Set reminders to take my seizure medications.	Take medicine with daily activities (breakfast, dinner, during TV show, before bed) Use calendar or set a daily reminder on your phone Use electronic reminders, text or email (My Epilepsy Diary or Texting 4 Control) Use a pill box
Lifestyle management (*n* = 5)^4^		
I rarely get enough sleep.	Get an average of 6–8 h of sleep every night.	Regulate sleeping habits
I never wear a bracelet / necklace or carry information stating that I have seizures / epilepsy	Always carry an ID stating your epilepsy	Get a medical alert device Wear a bracelet or necklace with health information Always carry ID with up-to-date information Other “None”
I usually go swimming alone	Do not go swimming alone	Other “None”
I never keep track of the side effects of my seizure medicine.	Keep a record of all the medication that you take and any of their side effects	Other “I do not see how those things would help with side effects. I do not even know which side effects come from which meds or which are due to my conditions.”
I never check with my doctor before taking other medicines.	Check with my doctor before taking other medications	Ask pharmacist about hazardous interactions between medicines

1 Each goal was selected by a single participant, except for ‘I never wear a bracelet/necklace or carry information stating that I have seizures/epilepsy’, which was selected by two participants.

2 Self-efficacy ratings were “very sure” for all goals. The exception was a rating of “not sure” for the goal of ‘getting an average of 6–8 h of sleep every night.’ (Coding note: Self-efficacy ratings were coded as binary within the MINDSET prototype.) A rating of ‘sure’ or ‘very sure’ was coded as “very sure” on their Action Plan; rating of ‘not sure’ or ‘somewhat sure’ was coded as “not sure” on their Action Plan).

3 When ‘Other’ was selected from the list of answer choices, the patient could then provide a written response in the text box provided.

4 ESM Domain; *n* = total # patients flagged as having a risk behavior for that domain. Participants who had no behaviors flagged as needing work were given a “Congratulations!” message for that domain instead of goals to choose from and were not included in this table.

### 3.3 Recommended ESM programs

None of the participants reported past exposure to any other MEW program (UPLIFT, PACES, or HOBSCOTH; Table 4, Column 4). The MINDSET 2.0 recommendations to participants for these programs (Table 4, Columns 5–7) corresponded to predicted recommendations based on behavioral, depression, and cognitive function metrics (Table 2, Columns 1–3) and a participant’s prior exposure to the programs (Table 4, Column 4). The MINDSET 2.0 recommendation algorithms demonstrated 100% agreement between programmed and generated recommendations, confirming that the software executed the predefined decision rules as intended. Clinical feasibility of MINDSET 2.0 was evaluated separately through usability, acceptability, and implementation-related measures. Among participants who reviewed the recommended MEW programs, 17 and 33% indicated that the programs appeared appropriate and helpful, respectively.

**Table 4:** ESM program recommendation algorithm functionality (*N* = 7).

Data input into my epilepsy										Data output (Action Plan)						Correspondence of predicted to actual ^6^
Participant	ESM behavior adherence flagged^1^			Depression^2^	Memory^3^		MEW previous exposure^4^			MEW program recommendation^5^						
										PACES		UPLIFT		HOBSCOTCH		
	S	M	L	(NDDI-E)	(QoLIE-10)	(QoLIE-31)	P	U	H	Predict	Action plan	Predict	Action plan	Predict	Action plan	
1	X	X	**✓**	X	X	X	No	No	No	**✓**	**✓**	X	X	X	X	100%
2	**✓**	**✓**	**✓**	**X**	**✓**	**✓**	No	No	No	**✓**	**✓**	X	X	**✓**	**✓**	100%
3	X	**✓**	**✓**	X	X	X	DK	DK	DK	**✓**	**✓**	X	X	X	X	100%
4	X	X	**✓**	X	X	X	No	No	No	**✓**	**✓**	X	X	X	X	100%
5	**✓**	**✓**	**✓**	X	**✓**	**✓**	DK	No	No	**✓**	**✓**	X	X	**✓**	**✓**	100%
6	X	X	**✓**	X	X	X	No	No	No	**✓**	**✓**	X	X	X	X	100%
7	X	X	X	X	X	X	No	No	No	**✓**	**✓**	X	X	X	X	100%

1 ESM Behaviors – an ESM domain (Seizure Management, Medicine Management, Lifestyle Management) is flagged as needing work if the patient reports at least one at-risk behavior within that domain.

2 Depression is indicated on the Action Plan if the patient obtains a summed score of 15 or more on the NDDI-E.

3 Cognitive Issues are indicated on the Action Plan if the patient obtains a score of 70 or lower on the QoLIE-31 cognitive subscale OR answers 3, 4, or 5 (extremely bothersome) on the QoLIE-10 item about memory OR both.

4 Past exposure to MEW Network programs is a criterion used to determine whether a patient receives a recommendation for one or more programs. Patients self-reported any past exposure as Yes (Y), No (N), or do not know (DK) for each of the three MEW programs.

5 Predicted (predict) and actual (AP) recommendations for MEW Network programs are shown (**✓** = recommended; X = not recommended).

6 Correspondence of predicted to actual recommendations. The benchmark is 100%.

### 3.4 Patient perceptions on usability

One patient was unable to complete the usability survey and so was not included in the usability analysis. As a result, the final sample size for usability analysis was six. When given the choice of English or Spanish versions of MINDSET 2.0, most patients selected the English version (85.7%). English language preference varied by state (AZ: 100%; TX: 66.7%). Participants’ ratings of usability exceeded the *a priori* threshold of 70% agreement for most parameters (Table 5). Participants agreed that MINDSET 2.0 was acceptable, likable, credible, easy to use, understandable, of appropriate duration, and appealing (100% agreement). They also rated MINDSET 2.0 as impactful in improving the thinking, discussion, and management of epilepsy (75–100%). Participants reported high perceived usefulness of MINDSET 2.0, with 100% indicating that the information provided by the tool was helpful for their epilepsy self-management. Participants were less sure if the recommendations of other MEW programs were helpful (33%) and if these programs were appropriate (17%), with most reporting that they did not know. Participants reported that it was easy to enter responses and choose goals and strategies (100%). Most (67%) reported that it was easy to understand and download the Action Plan PDF. Three participants (50%) indicated that they needed help with MINDSET 2.0.

**Table 5:** Patient agreement on usability parameters (*N* = 6)^1^.

Usability parameters	Items		Agreement N(%)
Acceptability	Entire program		6 (100)
Likeability^2^ (interface design)	Colors used in this program Buttons and sliders used in this program Size of the letters Layout of the screen		6 (100) 6 (100) 6 (100) 6 (100)
Motivational Appeal	I would recommend MINDSET 2.0 to other patients: Yes I would use MINDSET 2.0 again in a clinic visit: Yes^3^		6 (100) 4 (66.7)
Credibility	I think the information I got from MINDSET 2.0 can be trusted		6 (100)
Duration	*Time to use was*	Just right Too Long	5 (83.3) 1 (16.7)
Usefulness	I think the information I got from MINDSET 2.0 was helpful The MEW Network program(s) that MINDSET 2.0 suggested is appropriate for my needs^4^ Having MINDSET 2.0 suggest MEW Network program(s) was helpful MINDSET 2.0 is as useful or more useful than other seizure programs that I have used^5^		6 (100) 1 (16.7) 2 (33.3) 2 (33.3)
Perceived Impact	*I think the information provided…:*	Helped me to think carefully about my epilepsy. Will help me talk to my doctor or nurse about my epilepsy. Will help me manage my epilepsy better	5 (83.3) 6 (100) 5 (83.3)
Easy to Use^6^	It was easy to: Enter your responses into MINDSET 2.0 I think MINDSET 2.0 was easy to use		5 (83.3) 6 (100)
Understandability	I did not need help to answer questions I knew and understood most of the words		3 (50.0) 6 (100)
	*Action Plan development online*	Understand instructions for “My Goals” Understand the Action Plan It was easy to choose a goal to work on Choose your strategies	5 (83.3) 6 (100) 6 (100) 6 (100)
	*Action Plan development*	Understanding the PDF version of the action plan Understanding the PDF Action Plan layout Understanding the PDF Action Plan content Downloading PDF version of Action Plan^7^	4 (66.7) 4 (66.7) 4 (66.7) 3 (50.0)

1 Total sample is *N* = 7 but usability data for 1 participant is missing.

2 Likert scale: Like a lot (1)–Dislike a lot (4); Like = ‘Likes a lot’ and ‘like’.

3 Respondents who said do not know (*n* = 2).

4 Respondents who said do not know (*n* = 4).

5 Respondents who have not used another program for comparison (*n* = 4).

6 Likert scale: Very easy–Not easy; Easy = ‘somewhat easy’ and ‘very easy’.

7 Respondents who said do not know (*n* = 3).

The exit interview data supported the feasibility and usability ratings. The strengths of MINDSET 2.0 were that it provided a formalized approach to patient adherence and lifestyle issues, focused the patient on specific issues, was facilitated by a self-directed Action Plan, and enabled goal-setting that provided patients with strategies to work on.

### 3.5 Neurologist perspectives on MINDSET 2.0 implementation

The participating clinical neurologists have more than 10 years of clinical practice experience and over 5 years in their current practices. Overall, neurologists expressed positive perceptions of MINDSET 2.0, although their ratings varied across individual respondents (Table 6). All three neurologists viewed the tool favorably for improving the thoroughness of information collected and facilitating communication with patients, while perceptions differed regarding the impact on the ease of the clinical encounter, the accuracy of the assessment, and the time required to complete the visit. Neurologists commented that MINDSET 2.0 enabled discussions of issues that might not otherwise have been identified during routine clinic visits and reported that patients generally responded positively to the program, particularly those who recognized opportunities to improve their epilepsy self-management. They also reported that having patients identify their own goals and self-management strategies enhanced patient autonomy, encouraged greater engagement in their care, and increased attention to lifestyle management. All neurologists acknowledged that incorporating MINDSET 2.0 increased the duration of clinic visits and suggested several strategies to facilitate implementation, including asking patients to arrive approximately 30 min before their appointment, limiting the number of MINDSET 2.0 patients scheduled during a clinic session, dedicating specific clinic sessions for MINDSET 2.0 assessments, or allowing patients to complete MINDSET 2.0 and the associated Action Plan online before their scheduled visit.

**Table 6:** Neurologist ratings of MINDSET 2.0 (*N* = 3)^1^.

Using MINDSET 2.0 in my clinic made the encounter with my patient … ^2^	HCP #1	HCP #2	HCP #3	Mean	SD
Easier	3	3	5	3.67	1.15
Thorough	4	5	5	4.67	0.58
Accurate	3	3	5	3.67	1.15
Communicative	4	5	5	4.67	0.58
Feature 1: Using MINDSET 2.0 in my clinic made identifying my patient’s epilepsy self-management problems.					
Easier	3	5	5	4.33	1.15
Thorough	3	5	5	4.33	1.15
Accurate	3	5	5	4.33	1.15
Feature 2: Using MINDSET 2.0 in my clinic made determining my patient self-management goals and strategies.					
Easier	3	5	5	4.33	1.15
Thorough	3	5	5	4.33	1.15
Accurate	3	5	5	4.33	1.15
Feature 3: Using MINDSET 2.0 in my clinic made identifying my patient’s self-management barriers.					
Easier	3	5	5	4.33	1.15
Thorough	2	5	5	4	1.73
Accurate	2	5	5	4	1.73
Feature 4: Using MINDSET 2.0 in my clinic made identifying patients who need more intensive intervention and recommending specific programs for them.					
Easier	2	5	5	4	1.73
Thorough	2	5	5	4	1.73
Accurate	2	5	5	4	1.73
Feature 5: Using MINDSET 2.0 in my clinic made identifying patients’ negative social situations and providing resources to help alleviate those situations.					
Easier	3	5	5	4.33	1.15
Thorough	3	5	5	4.33	1.15
Accurate	3	5	5	4.33	1.15

1 One neurologist was not available to complete the survey.

2 Response set: 5-point Likert scale with semantic differential anchors (harder to do/easier to do, less thorough/more thorough, less accurate/more accurate, less communicative/more communicative).

### 3.6 Feasibility criteria and progression to future evaluation

Based on the prespecified feasibility criteria, MINDSET 2.0 demonstrated adequate feasibility to support further evaluation in a future randomized controlled trial. The recommendation algorithm demonstrated 100% agreement with predefined decision rules, exceeding the prespecified 70% threshold, and findings related to usability, acceptability, and workflow integration provided preliminary support for continued evaluation. Completion times indicated that integrating a comprehensive epilepsy self-management assessment into routine neurology practice may require extending clinic visits by approximately 30 min, consistent with recommendations from participating neurologists.

## 4 Discussion

This study assessed online decision support (MINDSET 2.0) to assess ESM behaviors and non-medical drivers of health and provide recommendations for evidence-based MEWN training programs to improve the management of epilepsy and related comorbidities. Participants’ reported ESM competencies and goal selection were consistent with previous studies of MINDSET and also consistent with the finding that MINDSET 2.0 was acceptable, useful, and appealing (15–18). These results suggest that enhancements to the original MINDSET 2.0 program did not degrade its acceptability by the patients.

Using the feasibility framework proposed by Bowen et al., findings from this study suggest that MINDSET 2.0 demonstrates feasibility across key domains of acceptability, demand, implementation, and practicality. The prespecified progression criteria were achieved, including successful execution of the recommendation algorithm, favorable usability ratings, and integration into the neurology clinic workflow. However, findings also identified areas requiring further refinement prior to an RCT, including strategies to improve patient engagement with recommended epilepsy self-management programs and optimization of clinic scheduling considerations.

The 100% correspondence between the recommendations generated by MINDSET 2.0 and the programmed decision rules demonstrates that the software successfully executed the predefined recommendation algorithm. However, these findings represent software verification and should not be interpreted as evidence of clinical effectiveness, appropriateness, or patient engagement with the recommended interventions. Participants’ ratings of the helpfulness and appropriateness of the recommended MEW training programs should also be interpreted with caution, as none of the participants had prior experience with MEW interventions. These ratings likely reflected participants’ initial perceptions based on the information provided within MINDSET 2.0 rather than informed evaluations of the programs’ usefulness or suitability. Nonetheless, these findings highlight the importance of incorporating strategies to enhance motivation, address perceived barriers, and facilitate enrollment in recommended self-management programs. Future studies should evaluate perceptions of MEW programs after participants have had direct exposure to or completed recommended interventions to better assess their relevance, usefulness, and acceptability.

The study addresses a need beyond assessment and feedback of patient ESM behaviors by also examining a potential strategy to increase the reach of MEWN programs. The feasibility and efficacy of enhanced decision support and action planning are predicated on a clinical infrastructure that is equipped to respond. This study focused on a single visit that established acceptance for the use of MINDSET 2.0 but did not assess subsequent linkage, enrollment, and retention in the recommended MEWN programs. MINDSET 2.0 is a useful assessment and feedback tool for PWE to improve ESM practice in neurology clinics, but its potential as a referral ‘hub’ to MEWN programs (PACES, UPLIFT, and HOBSCOTCH) and social services can only succeed if such services are available. These downstream functions are reflective of the availability of the programs and the operations of the clinic to ensure continuity and follow-through of care and will be important for subsequent investigation of efficacy of MINDSET 2.0.

The complexity of managing epilepsy has been well documented. PWE often require ongoing health and social care throughout their lifetime. Therefore, there is a recognized need to integrate care between different epilepsy services and across sectors (30, 31). Gandy et al. define integrated care as “an approach to health care that promotes coordination across multiple aspects of a person’s health care needs and may include linking services that are traditionally siloed” (31). The implementation of integrated care models is challenging because it requires standardized practice across healthcare systems and communication between providers (30). A recent scoping review identified shared decision-making, education for PWE and their HCPs, and linkage to psychosocial services among the top 15 interrelated components that were important for integrated epilepsy management. The integration of evidence-based MEWN programs as a component of integrated care is emerging (4, 32). Programs like MINDSET 2.0 may assist in increasing awareness of MEWN programs and how to utilize them effectively. The lack of MEWN program integration does not debilitate the utility of MINDSET 2.0 but undermines the potential benefits of the recommendation and NMDH assessment features.

Health care providers (HCPs) who implemented MINDSET 2.0 indicated that the Action Plan would have utility in neurology clinics, specifically in improving the thoroughness of data collection and patient communication. Although administration of the MINDSET 2.0 program required approximately 30 additional minutes to complete, it was successfully integrated into the existing neurology clinic workflow without disrupting routine clinical operations. These findings suggest that implementation is feasible when sufficient time is allocated for the self-management assessment. The MINDSET 2.0 features to assist HCPs in providing tailored recommendations to PWE on MEWN ESM programs appropriate to their needs and to alert them to non-medical drivers of health is consistent with IOM recommendations to strengthen an ecological approach to PWE that accommodates lifestyle and social welfare along with clinical services (33). Anchoring assessments and recommendation algorithms on validated and reliable scales can reinforce a data-driven approach to treatment planning that is accurate and parsimonious.

Feasibility assessment established the capability of MINDSET 2.0 to be accessed from different devices. Digital health tools have been strongly recommended for inclusion in integrated care models to support HCPs and their patients, but very few have been implemented and assessed (30). The recent upsurge in telemedicine and distance consultations also underscores the importance of accommodating patient needs at a distance. Web-based applications have the potential to combine the tailored approach of face-to-face clinical interventions with the scalability of public health interventions, and patients appreciate the accessibility and convenience. With access to technology soaring and the digital divide lessening, MINDSET was redesigned as a web-based application (MINDSET 2.0) in response to calls for a digital version of ESM. Although MINDSET 2.0 is accessible from any Internet-compatible device, it has yet to be integrated into clinic electronic health record systems. Reducing the use of dedicated devices could further streamline clinical experience.

The study findings need to be interpreted in light of several limitations, including a limited sample of PWE who were predisposed to volunteer for an ESM study and may not be representative of the broader population of PWE. The small sample size limits the precision and interpretability of percentage-based feasibility metrics. Given that each participant represents approximately 17% of the sample, a single participant response could substantially influence whether a predefined threshold was achieved. Therefore, these findings should be interpreted as preliminary indicators of feasibility that require confirmation in a larger sample prior to broader implementation or evaluation in a randomized controlled trial.

An additional limitation was the inability to fully evaluate recruitment feasibility. Although MINDSET 2.0 was implemented across multiple neurology clinics, no systematic tracking data on the total number of patients approached or reasons for declining participation were available. Therefore, we are unable to determine recruitment yield or fully characterize barriers to enrollment. Future feasibility studies should incorporate standardized recruitment tracking procedures, including documentation of patients screened, approached, eligible, enrolled, and reasons for non-participation, to better inform recruitment strategies for a future randomized controlled trial.

Although MINDSET 2.0 was designed as a bilingual tool to support accessibility among diverse populations, the majority of participants selected the English version (85.7%), and only one participant used the Spanish-language version. Therefore, this study provides limited evidence regarding the usability, acceptability, and cultural appropriateness of the Spanish version. Future evaluations should intentionally recruit Spanish-speaking individuals with epilepsy to assess the feasibility of the Spanish-language version and identify opportunities for further linguistic or cultural adaptation.

Despite strong feasibility precedent from previous MINDSET studies, this single-group cross-sectional study did not assess the functionality of MINDSET 2.0 across multiple clinic visits or track patients’ use of recommended MEWN programs or community services. Future studies should assess the longitudinal feasibility and efficacy of this approach. The MINDSET 2.0 algorithms are confined to recommendations for a limited number of MEWN ESM programs and do not include other evidence-based programs (SMART, TIME). The time-on-task and lack of integration of MINDSET 2.0 may represent adoption barriers in this regard. The MINDSET 2.0 upgrades included updated software and added decision-support algorithms. Future enhancements could include artificial intelligence to move MINDSET 2.0 toward a more naturalistic, personalized, and responsive user experience.

## 5 Conclusion

MINDSET 2.0 extends the functionality of the original MINDSET program beyond collating epilepsy and ESM data for goal-based decision support to aid the patient and neurologist in deciding which evidence-based MEWN ESM training programs are salient for the patient based on the patient’s own data and which non-medical determinants of health might require social service intervention. Future studies are needed to assess the efficacy of the MINDSET 2.0 features.

## Data Availability

The raw data supporting the conclusions of this article will be made available by the authors, without undue reservation.
